# Pontine stroke in a patient with Chronic Progressive External Ophthalmoplegia (CPEO): a case report

**DOI:** 10.1186/s12883-023-03249-9

**Published:** 2023-06-14

**Authors:** Yazan Eliyan, Kourosh Rezania, Christopher M. Gomez, Kaitlin Seibert

**Affiliations:** 1 Pritzker School of Medicine, University of Chicago, Chicago, IL USA; 2 Department of Neurology, University of Chicago Medical Center, Chicago, IL USA

**Keywords:** Chronic progressive external ophthalmoplegia (CPEO), Mitochondrial disorders, Neuromuscular disorders, Hereditary myopathy, Novel mutation, Case report

## Abstract

**Background:**

Chronic progressive external ophthalmoplegia (CPEO) is a mitochondrial disease with slowly progressive bilateral ptosis and symmetric ophthalmoplegia due to a genetic mutation that results in defective oxidative phosphorylation. Common genes that are implicated in CPEO include *POLG*, *RRM2B*, *ANT1* and *PEO1*/*TWNK*. Here, we report a case of a patient diagnosed with CPEO caused by a novel mutation in *PEO*/*TWNK* after suffering a right pontine stroke.

**Case presentation:**

A 70-year-old man with history of chronic progressive bilateral ptosis and ophthalmoplegia, as well as similar ocular symptoms in his father and grandfather, presented with acute onset of right hemifacial weakness and dysarthria. Brain MRI revealed an acute ischemic stroke in the right dorsal pons. The patient did not experience diplopia due to severe baseline ophthalmoplegia. Creatine kinase was elevated to 6,080 U/L upon admission and normalized over the course of one week; electromyography revealed a myopathic process. Genetic testing revealed a novel mutation c.1510G > A (p. Ala504Thr) in a pathogenic “hot spot” of the C10ORF2 gene (*TWNK*/*PEO1*), which is associated with CPEO. The mutation appears to be deleterious using several pathogenicity prediction tools.

**Conclusions:**

This case report describes a patient with late-onset CPEO caused by a novel, likely pathogenic, mutation in the *TWNK* gene. Although the patient presented with a pontine stroke, it manifested with solely new onset facial palsy, as he had a severe underlying ophthalmoplegia secondary to his CPEO.

## Background

Chronic progressive external ophthalmoplegia (CPEO) represents a heterogenous spectrum of mitochondrial disorders that manifest clinically as slowly progressive bilateral ptosis and symmetric ophthalmoplegia. In some cases, weakness of the pharyngeal muscles can cause dysphagia. CPEO can present as an isolated myopathy or in conjunction with other neurologic symptoms such as hearing loss, neuropathy, and ataxia (termed CPEO +) [1]. The disease is more common in females (ratio 2.5:1), and it typically presents during mid-adulthood [2]. CPEO results from defective oxidative phosphorylation in mitochondria caused by: 1) mutations in nuclear DNA responsible for maintenance of mitochondrial DNA (mtDNA), 2) point mutations in mitochondrial transfer RNA genes, or 3) single, large deletions of mtDNA [1–6].

The nuclear genes associated with CPEO are *POLG/POLG2, PEO1 (TWNK), RRM2B, SLC25A4 (ANT1), DNA2, OPA1, TYMP, MGM1, RNASEH1, TK2, and DGUOK*; mutations in these genes are usually inherited in an autosomal dominant or autosomal recessive fashion [1–4, 7, 8]. The mitochondrial transfer RNA gene implicated in CPEO is *MT-TL1*; mutations in this gene are inherited in a mitochondrial pattern. Single, large deletions of mtDNA are typically sporadic; these sporadic events make up approximately half of the cases [1, 3]. Here, we report a patient presenting with CPEO caused by a novel mutation in *PEO1/TWNK*, who was diagnosed after he presented with right facial palsy due to a pontine stroke.

## Case presentation

**Fig. 1:**
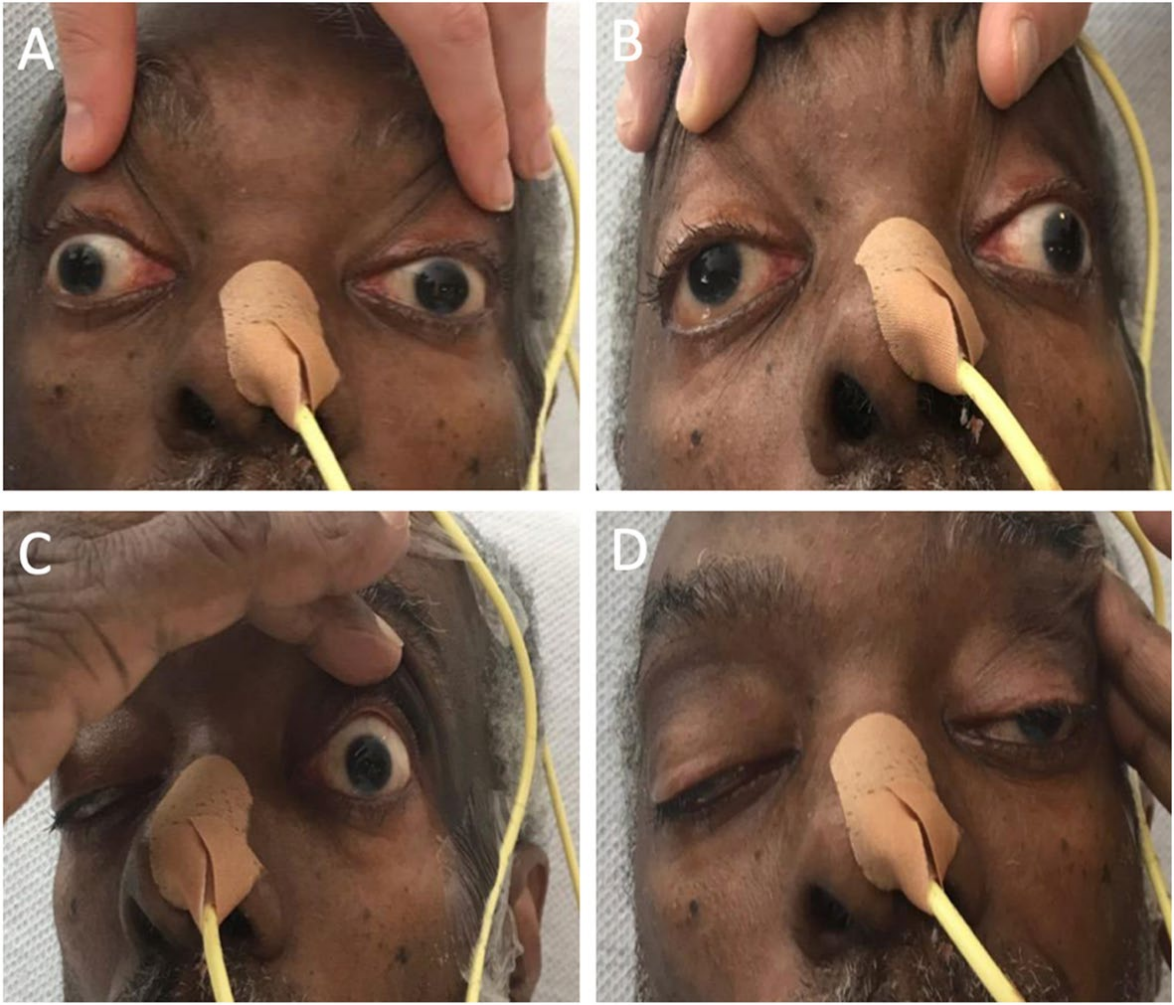
Bilateral ophthalmoplegia and ptosis. Severely restricted bilateral eye movement on rightward gaze (**A**), and leftward gaze (**B**); severe bilateral ptosis requiring physical lifting of eyelids in order for the patient to see (**C**-**D**)

A 70-year-old African American male presented to the emergency room after acute onset of right facial weakness and dysarthria for five hours. Demographic data is available in Table 1. On further questioning, he had non-fluctuating, progressive ptosis for approximately 10 years, as well as worsening dysphagia for liquids and solids over the previous several months. There was a family history of bilateral ptosis in his father and paternal grandfather. Detailed examination demonstrated symmetrical and bilaterally reactive pupils, severe bilateral ptosis and ophthalmoplegia (Fig. 1), with complete vertical gaze palsy and severely limited horizontal eye movements, and right-sided facial weakness involving the orbicularis oculi and oris. Convergence was impaired. Palatal elevation and swallowing were impaired. There was a complete vertical gaze palsy. There was no muscle weakness in the upper or lower limbs. Deep tendon reflexes were normal aside from absent ankle jerks. The patient denied pain.

**Table 1:** Demographic and laboratory data

***Demographic***		
Age	70	
Ethnicity	African American/Black	
Gender	Male	
***Laboratory Test***	***Patient Value***	***Normal Value***
Sodium (mmol/L)	137	134–149
Potassium (mmol/L)	4.5	3.5–5.0
Chloride (mmol/L)	98	95–108
Carbon Dioxide (mmol/L)	25	23–30
Anion Gap (mmol/L)	14	6–15
BUN (mg/dL)	15	7–20
Creatinine (mg/dL)	0.7	0.5–1.4
Calcium (mg/dL)	9.0	8.4–10.2
Inorganic Phosphate (mg/dL)	3.2	2.5–4.4
Magnesium (mg/dL)	2.0	1.6–2.5
Glucose (mg/dL)	170	60–99
AST (U/L)	21	8–37
ALT (U/L)	24	8–35
Lactic Acid (mmol/L)	1.1	0.7–2.1
Creatinine Kinase (U/L)	6,080, trended down to 477	9–185
LDL (mg/dL)	131	60–129
Hemoglobin A1c (%)	5.6	< 5.7
LDH (U/L)	347	116–245
TSH (mcU/mL)	0.56	0.30–4.00

*mmol/L* millimoles per liter, *mg/dL* milligrams per deciliter, *U/L* Units per liter, *mcU/mL* microunit per milliliter

**Fig. 2:**
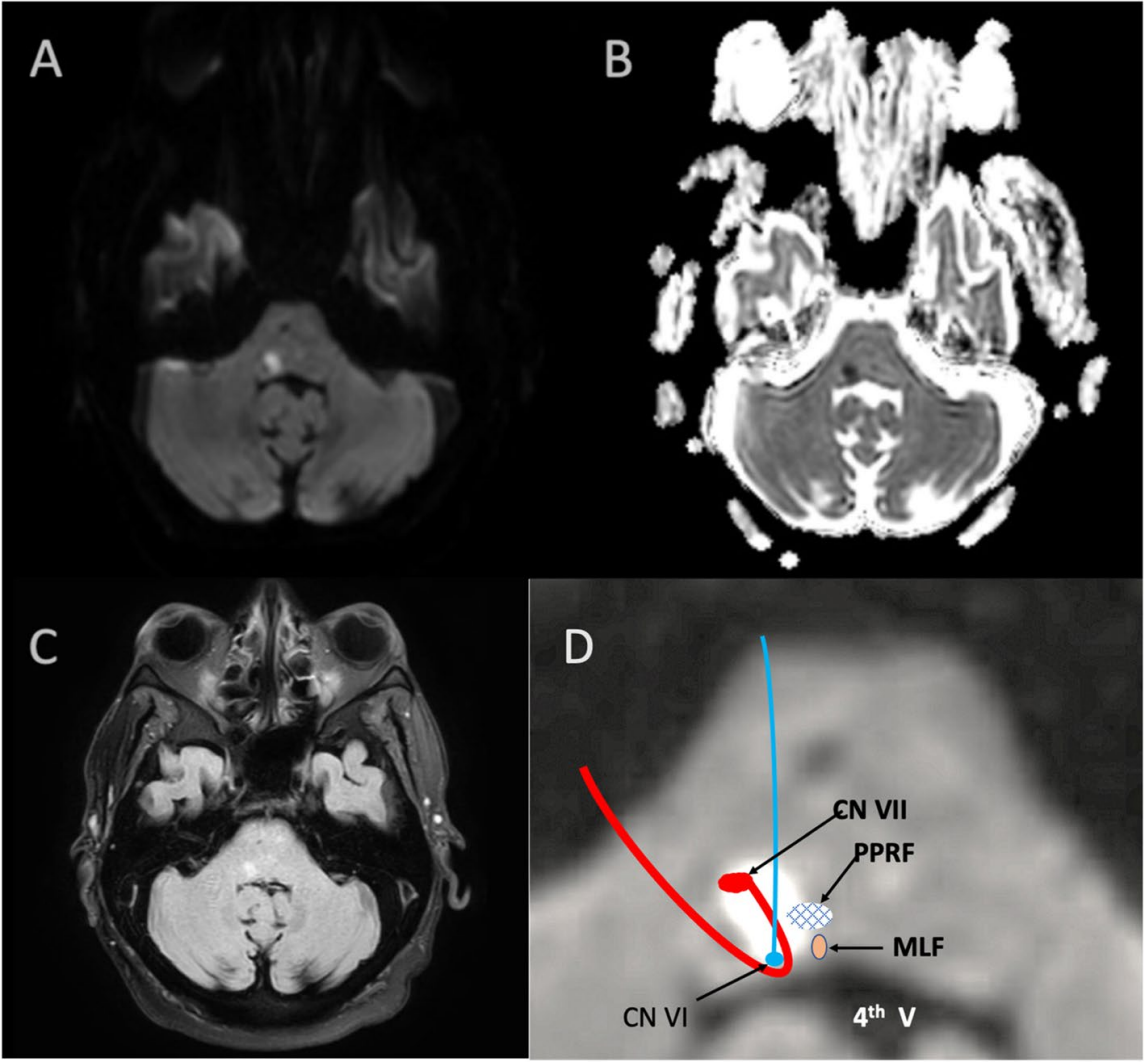
Pontine stroke on MRI Brain and diagram of affected and nearby structures. Axial sections through the pons demonstrate a lesion which was hyperintense on diffusion-weighted imaging (DWI) sequence (**A**), hypointense on apparent diffusion coefficient (ADC) sequence (**B**), and hyperintense on T2 and fluid attenuated inversion recovery (FLAIR) (**C**). The approximate location of facial and abducens nerves and related oculomotor structures are shown in image D. *V: ventricle, CN: cranial nerve, PPRF: parapontine reticular formation, MLF: medial longitudinal fasciculus*

Based on acuity of symptoms, an acute stroke was considered. A brain CT scan was obtained in the emergency department and was negative for hemorrhage. A brain MRI the following day revealed an acute ischemic stroke in the right dorsal pons at the level of the seventh cranial nerve nucleus and tract (Fig. 2). The patient presented out of the thrombolysis window, so the ischemic stroke was managed with secondary prevention measures. Initial relevant lab work (Table 1) revealed elevated creatine kinase (CK) to 6,080 U/L (decreased to 477 U/L over a week), borderline high low-density lipoprotein (LDL) (131 mg/dL), and normal hemoglobin A1c (5.6%).

A nerve conduction study, in conjunction with absent ankle reflexes, suggested a possible length-dependent sensory axonal neuropathy (Table 2). The compound muscle action potentials (CMAPs) were single-peaked. Repetitive nerve stimulation of the left facial nerve (recorded at nasalis) and left spinal accessory nerve (recorded at trapezius) did not show any decrement of the CMAP amplitudes. Neither oral pyridostigmine nor an ice pack test resulted in improvement of oculobulbar symptoms. Serum antibodies against acetylcholine receptors and muscle specific tyrosine kinase were negative.

**Table 2:** Nerve conduction study results for EMG

Nerve (unit)	Normal value	**Patient value**
**Right Median**		
Motor distal latency (ms)	< 4.1	3.33
CMAP amplitude- wrist (mV)	> 5.0	10.8
CMAP amplitude- elbow (mV)	-	10.4
Motor conduction velocity (m/s)	> 48	57.4
F wave latency (ms)	< 30	27.45
**Right Peroneal**		
Motor distal latency (ms)	< 6.0	4.90
CMAP amplitude- ankle (mV)	> 3.0	7.1
CMAP amplitude- fibular head (mV)	-	5.6
Motor conduction velocity (m/s)	> 38	49.3
F wave latency (ms)	< 57	49.53
**Right Radial**		
SNAP amplitude (μV)	> 15.0	17.3
SNAP conduction velocity (m/s)	> 50	51.5
**Right sural**		
SNAP amplitude (μV)	> 5.0	NR
SNAP conduction velocity (m/s)	> 38	-

*CMAP* compound motor action potential, *SNAP* sensory nerve action potential, *ms* millisecond, *mV* millivolt, *μV* microvolt, *m/s* meter per second, *NR* no response

Needle electromyography (EMG) showed fibrillation potentials and positive waves in all muscles tested in the right upper and lower limb and thoracic paraspinals; motor unit action potentials were overall normal and motor unit recruitment was normal in all muscles except the right deltoid, which was myopathic. Cerebrospinal fluid (CSF) analysis showed normal white blood cell count and protein levels and negative cytology. The following tests were negative or normal: CT scan of chest/abdomen/pelvis, serum and urine protein immunoelectrophoresis and immunofixation, and serum and CSF lactate and angiotensin converting enzyme levels. The patient declined a muscle biopsy.

**Table:** 

EMG Summary Table									
	**Spontaneous**					**MUAP**			**Recruitment**
	**IA**	**Fib**	**PSW**	**Fasc**	**Other**	**Amp**	**Dur**	**Morp**	**Pattern**
**R. DELTOID**	**Inc**	None	**1 + **	None	None	N	N	N	**early**
**R. BICEPS**	N	**1 + **	**1 + **	None	None	N	N	N	**N**
**R. FLEXOR CARPI RADIALIS**	**Inc**	**1 + **	**1 + **	None	None	**N**	N	N	**N**
**R. 1**^**ST**^** DORSAL INTEROSSEOUS**	**Inc**	**1 + **	None	None	None	N	N	N	**N**
**R. TIBIALIS ANTERIOR**	**Inc**	**1 + **	**1 + **	None	None	N	N	N	**N**
**R. VASTUS MEDIALIS**	**Inc**	None	**1 + **	None	None	N	N	N	**N**
**R. THORACIC PARASPINALS**	Inc	2 +	2 +	None	None				

*MUAP* motor unit action potential, *IA* insertional activity, *Fib* fibrillation potentials, *PSW* positive sharp waves, *Fasc* fasciculation potentials, *Amp* amplitude, *Dur* duration, *R* right, *Inc* increased, *N* normal

Based on the suspicion of a mitochondrial disorder and the absence of a viable tissue biopsy for biochemical and histochemical analysis, genetic testing was completed in the blood. Whole-exome sequencing (WES), as opposed to multi-gene panel or targeted next-generation sequencing approaches, was pursued given its emergence as a preferred and more effective diagnostic tool for suspected mitochondrial disease and other mimic neuromuscular disorders such as congenital myasthenias [9–11]. The success of WES compared to other approaches stems from the significant genetic and clinical heterogeneity of mitochondrial disorders and of neuromuscular disorders that can have overlapping clinical features [9].

WES demonstrated a variant (c.1510G > A (p.Ala504Thr) in the *PEO1*/*TWNK* (C10ORF2, NM_021830.4/5) gene, which encodes a mitochondrial protein known as “twinkle,” a DNA helicase involved in maintaining mtDNA [12]. The p.Ala504Thr substitution is likely deleterious using several in-silico pathogenicity prediction tools: Sorting Intolerant From Tolerant (SIFT) [13], PolyPhen2 [14], Align Gradient Validation Gradient Deviation (GVGD) [15], and Rare Exome Variant Ensemble Learner (REVEL) [16]. As such, this mutation represents a novel mutation associated with CPEO.

After the patient was discharged from the hospital, he was lost to follow-up; and genetic testing could not be completed on any of the patient’s affected family members as they were deceased.

## Discussion and conclusions

Clinically, this patient’s presentation of both an acute pontine stroke and CPEO presents several interesting learning points. First, it is rare for a pontine stroke to cause an isolated CN VII palsy. If the patient did not have baseline ophthalmoplegia, his stroke would have caused impaired right eye abduction due to right abducens palsy, impaired right eye adduction due to involvement of right medial longitudinal fasciculus, and impaired left eye adduction due to involvement of parapontine reticular formation (i.e. one and a half syndrome). In addition to one and a half syndrome, the stroke would have also caused a right facial palsy due to involvement of CN VII, as shown in Fig. 2D [17]. However, his stroke-related oculomotor symptoms were obscured by his underlying ophthalmoplegia. Second, stroke or stroke-like episodes are potential manifestations of mitochondrial disease, classically in the context of mitochondrial encephalomyopathy, lactic acidosis, and stroke like symptoms (MELAS). Our patient did not have the MELAS phenotype or a known mutation associated with MELAS, and the location of stroke was uncharacteristic for strokes associated with MELAS, which are typically cortical or thalamic and may not conform to any known vascular territory [18]. On the other hand, it has been proposed that mitochondrial disease aside from MELAS may predispose to stroke [19]. Considering his age, presence of small vessel disease and hypertensive angiopathy on brain imaging and range of elevated blood pressures while admitted, the etiology of his pontine lacunar stroke was likely small vessel disease. Computed Tomography Angiography (CTA) of the head and neck demonstrated diffuse atherosclerotic disease, including multifocal stenosis of internal carotid arteries, vertebral arteries and bilateral middle cerebral arteries. Based on time course, the constellation of presenting symptoms were separated based on acuity. The patient experienced sudden-onset right hemifacial paralysis and dysarthria. The weakness of upper and lower face in this patient suggested a lower motor neuron lesion, involving the facial nerve nucleus, fasciculus or nerve. A CVA involving cranial nerve VII nucleus or its tract was considered, given the acute onset and age of the patient.

The differential diagnoses for this patient’s progressive symmetric oculobulbar weakness included: 1) neuromuscular junction (NMJ) disorders, specifically myasthenia gravis and congenital myasthenic syndromes; and 2) heredofamilial myopathies, such as CPEO, oculopharyngeal muscular dystrophy, and myotonic dystrophy. In this patient, a neuromuscular junction disorder was considered unlikely based on negative repetitive nerve stimulation, ice pack test, and myasthenia gravis serologies. Normal configuration of CMAPs argued against a slow channel syndrome. The presence of diffuse fibrillations and positive waves in the EMG were suggestive of an underlying myopathy (rather than diffuse denervation) given the overall clinical presentation, markedly elevated CK, and presence of myopathic motor unit recruitment in the deltoid.

A mitochondrial myopathy explained not only the chronic oculobulbar symptoms but also the distal neuropathy and subclinical episode of rhabdomyolysis [20, 21]. Other heredofamilial myopathies, such as oculopharyngeal muscular dystrophy and myotonic dystrophy type I, were excluded with genetic testing. Although a muscle biopsy was not pursued in this case, the clinical characteristics are quite suggestive of a diagnosis of CPEO and align with the effects of similar mutations in the *PEO1*/*TWNK* gene.

*PEO1* (*TWNK*) is a nuclear gene that encodes for the twinkle protein and mutations in this gene are associated with CPEO. Autosomal dominant mutations in *PEO1* have been shown to cause a constellation of symptoms including ptosis, external ophthalmoplegia, and myopathy. Autosomal recessive mutations in *PEO1* have been implicated in infantile-onset spinocerebellar ataxia [22]. Both the clinical presentation and family history (similar symptoms present in patient’s father and paternal grandfather) are consistent with an autosomal dominant inheritance pattern; therefore, we did not proceed with mitochondrial DNA sequencing. The twinkle protein consists of 684 amino acids with a molecular mass of 77kD [12]. Van Hove et al. noted that the protein has three functional domains: a 5’ primase domain, a linker domain, and a 3’ helicase domain [22]. The majority of human mutations occur in the linker and helicase domains, and the pathogenic mutations in *PEO1* are clustered between residues Arg303 and Tyr508 [6, 22]. This patient’s novel mutation (p.Ala504Thr) is located in the helicase domain and affects residues that are predicted to be involved with stabilizing oligomeric structure [23, 24].

Our case study has several limitations: 1) we were unable to obtain a muscle biopsy, which may have further supported the diagnosis of a mitochondrial myopathy, and 2) genetic testing could not be performed in the patient’s family members to confirm the familial inheritance and pathogenic nature of the mutation.

To summarize, we present a 70-year-old male with acute onset right hemifacial weakness superimposed on chronic progressive bilateral ptosis, ophthalmoplegia, and dysphagia. The time course of the symptoms and proper anatomic localization were integral in recognizing the presence of multiple pathologies: a pontine stroke with an acute onset, isolated cranial nerve VII palsy, and an underlying CPEO, a mitochondrial myopathy with chronic and progressive oculobulbar weakness. Genetic testing revealed a novel variant in a pathogenic “hot spot” of the PEO1 gene, supportive of the diagnosis of CPEO.

## Data Availability

The principal data gathered and analyzed during this study are included in this published article. Data are available from the corresponding author on reasonable request.

## Abbreviations

CPEO: Chronic Progressive External Ophthalmoplegia
DNA: Deoxyribonucleic Acid
RNA: Ribonucleic Acid
mtDNA: Mitochondrial Deoxyribonucleic Acid
CT: Computed Tomography
MRI: Magnetic Resonance Imaging
CK: Creatine Kinase
LDL: Low-density Lipoprotein
DWI: Diffusion-Weighted Imaging
ADC: Apparent Diffusion Coefficient
FLAIR: Fluid Attenuated Inversion Recovery
V: Ventricle
CN: Cranial Nerve
PPRF: Parapontine Reticular Formation
MLF: Medial Longitudinal Fasciculus
CMAP: Compound Muscle Action Potential
EMG: Electromyography
CSF: Cerebrospinal Fluid
WES: Whole-exome sequencing
SIFT: Sorting Intolerant From Tolerant
GVGD: Align Gradient Validation Gradient Deviation
REVEL: Rare Exome Variant Ensemble Learner
MELAS: Mitochondrial Encephalomyopathy, Lactic Acidosis, and Stroke Like Symptoms
CVA: Cerebrovascular Accident
NMJ: Neuromuscular Junction
kD: Kilodalton
